# A novel method based on two cameras for accurate estimation of arterial oxygen saturation

**DOI:** 10.1186/s12938-015-0045-1

**Published:** 2015-05-30

**Authors:** He Liu, Kamen Ivanov, Yadong Wang, Lei Wang

**Affiliations:** Biomedical Engineering Department, Harbin Institute of Technology, Harbin, 150001 China; Shenzhen Key Laboratory for Low-cost Healthcare, Key Lab for Health Informatics, Shenzhen Institutes of Advanced Technology, Chinese Academy of Sciences, Xueyuan Avenue 1068, Shenzhen, 518055 China.

**Keywords:** Photoplethysmographic imaging, Arterial oxygen saturation, Pulse oximetry, Smoothed pseudo Wigner–Ville distribution

## Abstract

**Background:**

Photoplethysmographic imaging (PPGi) that is based on camera allows acquiring photoplethysmogram and measuring physiological parameters such as pulse rate, respiration rate and perfusion level. It has also shown potential for estimation of arterial oxygen saturation (SaO_2_). However, there are some technical limitations such as optical shunting, different camera sensitivity to different light spectra, different AC-to-DC ratios (the peak-to-peak amplitude to baseline ratio) of the PPGi signal for different portions of the sensor surface area, the low sampling rate and the inconsistency of contact force between the fingertip and camera lens.

**Methods:**

In this paper, we take full account of the above-mentioned design challenges and present an accurate SaO_2_ estimation method based on two cameras. The hardware system we used consisted of an FPGA development board (XC6SLX150T-3FGG676 from Xilinx), with connected to it two commercial cameras and an SD card. The two cameras were placed back to back, one camera acquired PPGi signal from the right index fingertip under 660 nm light illumination while the other camera acquired PPGi signal from the thumb fingertip using an 800 nm light illumination. The both PPGi signals were captured simultaneously, recorded in a text file on the SD card and processed offline using MATLAB®. The calculation of SaO_2_ was based on the principle of pulse oximetry. The AC-to-DC ratio was acquired by the ratio of powers of AC and DC components of the PPGi signal in the time–frequency domain using the smoothed pseudo Wigner–Ville distribution. The calibration curve required for SaO_2_ measurement was obtained by linear regression analysis.

**Results:**

The results of our estimation method from 12 subjects showed a high correlation and accuracy with those of conventional pulse oximetry for the range from 90 to 100%.

**Conclusions:**

Our method is suitable for mobile applications implemented in smartphones, which could allow SaO_2_ measurement in a pervasive environment.

## Background

Adequate oxygen supply to the body’s tissues is required for normal body function. The arterial oxygen saturation (SaO_2_) is an important physiological parameter which could be helpful in assessing heart and lung health, proper blood flow, and blood related issues. Pulse oximetry which was first invented by Aoyagi et al. [1] in 1974 is a noninvasive technique to monitor SaO_2_. At the same time, it also allows acquiring photoplethysmogram (PPG) and measurement of physiological parameters such as pulse rate (PR), respiration rate (RR) and perfusion level [2]. Pulse oximetry plays an important role in monitoring the health of patients. A pulse oximeter probe typically consists of two light-emitting diodes (LEDs) and a photodiode detector. The light can be reflected by the skin, or, more commonly, transmitted through the tissue. The probe is usually attached to an extremity (typically a finger or earlobe) by a plastic clamp that serves to maintain good contact with the tissue and to shield the detector from extraneous light sources.

In the past decades, photoplethysmographic imaging (PPGi) that is based on a camera for PPG signal acquisition became attractive due to its simplicity and noninvasiveness [3, 4]. The volumetric variation of blood changes the light absorption which allows PPGi evaluation with accuracy comparable with that achieved using the conventional PPG method when measuring parameters such as PR and RR [5, 6]. Some research groups have also explored the potential for estimation of SaO_2_ based on PPGi technology utilizing the principle of pulse oximetry. Two different approaches (i.e., contactless and direct-contact approaches) for estimation of SaO_2_ based on PPGi technology have been suggested. In terms of contactless approach, Wieringa et al. [7] first investigated contactless “SpO_2_ camera” technology which used multiple PPGi signals captured under light of different wavelengths. Humphreys et al. [8] have introduced a CMOS camera-based system for non-contact pulse oximetry imaging. The two studies showed that it is feasible to estimate oxygen saturation by means of contactless methods. Kong et al. [9] introduced a non-contact detection of SaO_2_ using ambient light for illumination and two CCD cameras, each one provided with a different narrow-band filter (i.e., 660 and 520 nm, respectively). Their result of SaO_2_ estimation showed a high correlation with that of the conventional pulse oximetry. However, it needed a complicated experimental arrangement. The second approach requires direct skin contact. A fingertip may be placed on the lens of a mobile phone digital camera. For instance, Scully et al. [10] presented an SaO_2_ measurement method based on comparing PPGi waveforms of the red and blue bands captured using reflection from the skin and a flash light for the illumination. In this case, the red and blue bands corresponded to the red and infrared wavelengths of conventional pulse oximeter, respectively. However, some technical limitations were not considered, such as the optical shunting, the different camera sensitivity to different light spectra, different AC-to-DC ratios (the peak-to-peak amplitude to baseline ratio) of the PPGi signal for different portions of the sensor surface area, the low sampling rate [11] and the inconsistency of contact force between the fingertip and camera lens [12].

In this paper, we focus on the direct-contact approach for estimation of SaO_2_, based on back-to-back placed cameras in a pervasive environment. We take full account of the above-mentioned design challenges. The back-to-back placed micro cameras were used to simultaneously acquire two PPGi signals from the right index and thumb fingertips of participants, respectively. One PPGi signal was obtained under 660 nm wavelength light illumination and the other was obtained under 800 nm wavelength light illumination. The calculation of SaO_2_ derived from the AC and DC components of PPGi signal was based on the principle of pulse oximetry. The AC-to-DC ratio was acquired from the ratio of the powers of PPGi signal AC and DC components in the time–frequency domain, using the smoothed pseudo Wigner–Ville distribution (SPWVD).

## System design

### Image sensor module

Two identical simple and inexpensive (<$10) digital cameras of type OV9715 (from OmniVision, Santa Clara, USA) were used as image sensors. OV9715 is a low-voltage and high-performance CMOS WXGA (1,280 × 800 pixels) camera which works at a fixed rate of 30 fps, and all its functional units are integrated into a single chip. It provides full-frame and windowed 8-bit/10-bit images in raw red, green and blue (RGB) format via Digital Video Port (DVP).

### Prototype system

**Figure 1:**
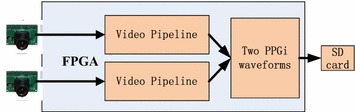
Block-diagram of the acquisition system used to obtain the two PPGi waveforms.

The hardware designed for this study was partly based on our previous work [13, 14]. The system consisted of an FPGA development board (XC6SLX150T-3FGG676 from Xilinx), two commercial cameras and an SD card. The cameras were connected to the FPGA development board. All features of each camera, including the exposure control, white balance control, defective pixel cancellation etc. were configured by commands sent from the development board to the camera through the serial camera control bus. By proper adjustment of the camera parameters, clear images were acquired. In the FPGA development board the raw data from the two cameras were first passed to the video pipeline and then the data of the extracted PPGi waveforms were recorded in a text file on the SD card (Figure 1). This text file was copied to a PC for the subsequent data analysis.

### Subjects and experimental protocol

**Figure 2:**
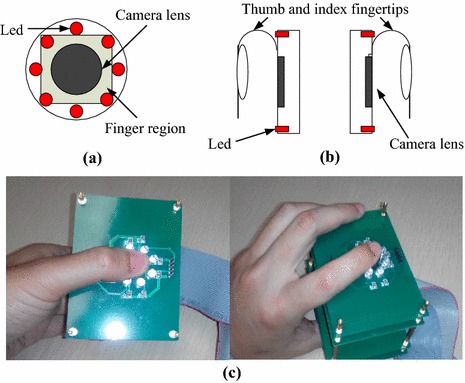
**a** Top view, **b** side view of the camera and **c** a real-world implementation using a smartphone mock-up.

A total of 12 healthy volunteers (10 males, aged from 24 to 35 with a mean age of 28.6 years and 2 females, both aged 25 years), recruited from the Shenzhen Institutes of Advanced Technology, Chinese Academy of Sciences, were enrolled in this study. None of them had any known cardiovascular or diabetic disease. For obtaining the reference SaO_2_, an FDA-approved SaO_2_ measurement system, TSD123 connecting to one OXY100C module from BIOPAC was used. The reference SaO_2_ was acquired at a sampling rate of 1 kHz. Each subject was required to avoid any body movements during image capture. Two narrow-band, accurate-wavelength ring LED illuminators (from EPITEX), with wavelength of 660 and 800 nm, respectively, were used for the illumination at the two ROIs. As shown in Figure 2a, each ring illuminator was attached to the lens of a digital camera. The light reflected from the skin surface of each of the two fingertips was collected into the corresponding monochrome CMOS camera (Figure 2b). The signal which reflects the cardiac rhythm was then extracted from the recorded video of each camera to estimate the AC and DC components. All experiments were conducted in a light-protected chamber. Since the temperature at the measurement sites also affects the AC and DC components of PPGi signal [15], a temperature sensor (TSD202D from BIOPAC) was used to record the temperature at the ROIs at the beginning and at the end of the experiment.

**Figure 3:**
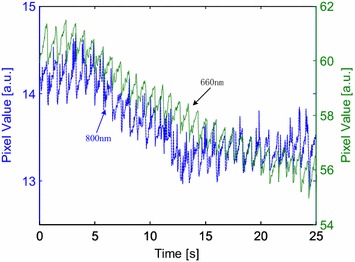
Two PPGi signals simultaneously recorded under 660 and 800 nm light illumination, respectively.

The experimental procedures were arranged into two consecutive stages and every stage consisted of three sessions. In session 1, each participant kept normal breathing and placed the two ROIs directly on the lens of the cameras, consciously excreting stable contact force for 30 seconds (s). Due to the nature of the experiment, the forces excreted over the two camera lens were equal in magnitude. In session 2, each participant was required to hold his breath for approximately 40 s. In session 3, the participants restored normal breathing for about 120 s. In stage 2, the subjects were required to exert higher contact force compared to that exerted in all sessions of stage 1. Figure 2b illustrates the experimental arrangement where the right index and thumb fingertips of participants were chosen as ROIs. Figure 2c shows a real-world implementation using a smartphone mock-up. Figure 3 shows two PPGi signals simultaneously recorded under 660 and 800 nm light illumination, respectively.

## Methods

### The principle of pulse oximeters

SaO_2_ indicates the ratio of oxygenated hemoglobin concentration (i.e., HbO_2_) to total hemoglobin concentration in the blood (i.e., HbO_2_ + Hb) which is defined as follows:

1 $$ SaO_{2} = \frac{{HbO_{2} }}{{HbO_{2} + Hb}} \times 100\% $$

The theory of pulse oximetry has been described in several studies [16–18]. PPG signals captured while alternately applying light illumination of two different wavelengths are recorded and SaO_2_ is derived from *R* which is defined as follows:

2 $$ R = \frac{{{{AC_{{\lambda_{1} }} } \mathord{\left/ {\vphantom {{AC_{{\lambda_{1} }} } {DC_{{\lambda_{1} }} }}} \right. \kern-0pt} {DC_{{\lambda_{1} }} }}}}{{{{AC_{{\lambda_{2} }} } \mathord{\left/ {\vphantom {{AC_{{\lambda_{2} }} } {DC_{{\lambda_{2} }} }}} \right. \kern-0pt} {DC_{{\lambda_{2} }} }}}} $$

where AC is the peak-to-peak amplitude (originating from the arterial blood pulsation) and DC is the baseline component (determined by the light-conducting/reflecting properties of venous blood, tissue, bones, etc.) of the measured PPG signal for the respective wavelengths $\lambda_{1}$ and $\lambda_{2}$. Using Eq. 2, the theoretical calibration curve for the calculation of functional SaO_2_ can be defined as follows:

3 $$ SaO_{2} = \frac{{\varepsilon_{Hb}^{{\lambda_{1} }} - \varepsilon_{Hb}^{{\lambda_{2} }} \times R}}{{\varepsilon_{Hb}^{{\lambda_{1} }} - \varepsilon_{{HbO_{2} }}^{{\lambda_{1} }} + (\varepsilon_{{HbO_{2} }}^{{\lambda_{2} }} - \varepsilon_{Hb}^{{\lambda_{2} }} ) \times R}} \times 100\% $$

where $\varepsilon_{i}$ is the extinction coefficient at a specific wavelength $\lambda$.

Generally, the relationship between $R$ and SaO_2_ is empirically determined by the linear regression curve and the linear regression function can be expressed as follows:

4 $$ SaO_{2} = \alpha_{1} \cdot R + \beta_{1} $$

where the y-intercept $\beta_{1}$ and the slope $\alpha_{1}$ are empirical coefficients determined by calibration.

### Light spectra and camera sensitivity

**Figure 4:**
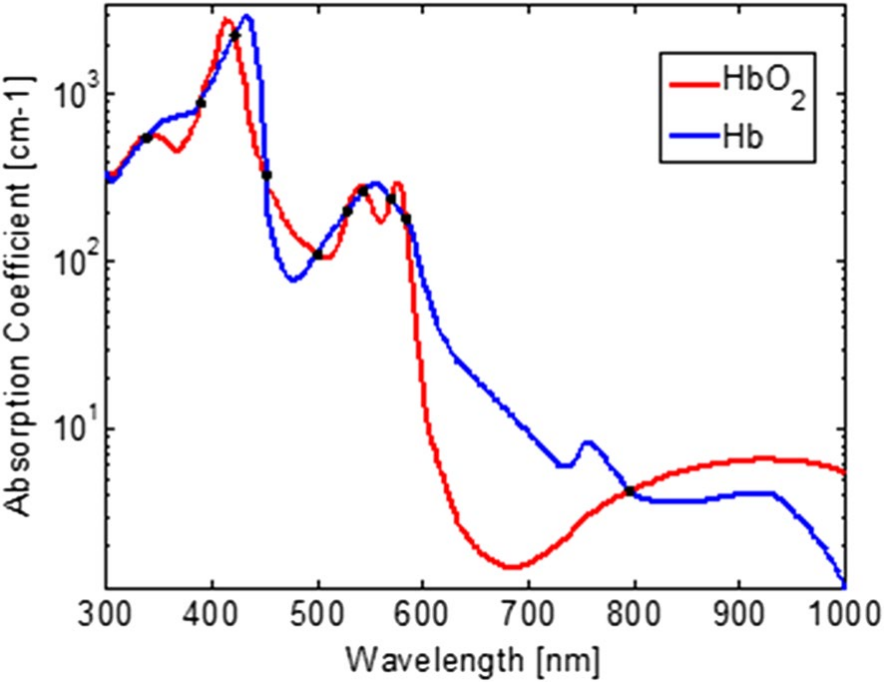
Absorption curves for HbO_2_ and Hb for light spectrum in the range from 300 to 1,000 nm. Created with data from [19]. *Black points* indicate equal absorption coefficients of HbO_2_ and Hb observed at wavelengths of 338, 390, 422, 452, 500, 528, 544, 584 and 800 nm, respectively.

**Figure 5:**
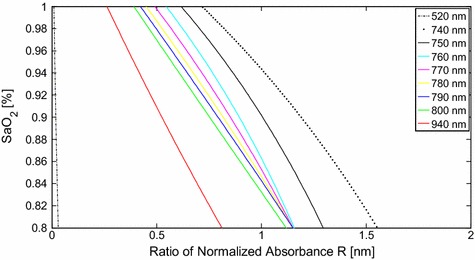
Theoretical relationship between R and SaO_2_ calculated according to Eq. 4 for several pairs of wavelengths, where one of the wavelengths in the pair is always 660 nm.

Conventional pulse oximetry relies on the fact that HbO_2_ and Hb have different absorption spectra, thus, analyzing PPG signals captured under lights of two different wavelengths allows for estimation of the SaO_2_ level. In this case the wavelength selection follows two principles. First, the absorption coefficients of HbO_2_ and Hb at the first wavelength should differ significantly. The wavelength of 660 nm was chosen as one of the two wavelengths in the conventional pulse oximeter due to the maximal difference between the absorption coefficients of HbO_2_ and Hb. Since the camera we used in our previous work [20] showed high sensitivity to 660 nm wavelength light, we chose 660 nm as one of the two light wavelengths for our proposed method. The second principle of conventional pulse oximetry is to ensure approximately equal absorption coefficients of HbO_2_ and Hb at the other wavelength. Infrared light with a wavelength in the range from 740 to 940 nm may be chosen as the second illumination light. Figure 4 shows the absorption curves for HbO_2_ and Hb for light spectrum in the range of 300 to 1,000 nm. The HbO_2_ and Hb absorption coefficients are equal not only for infrared light, but also for light with wavelength of 338, 390, 422, 452, 500, 528, 544 and 584 nm. In our previous study, we investigated the performance of PPGi signal acquisition from the RGB channels using a commercial camera under different monochromatic lights in the range of visible light [20]. Compared with the gold standard PPG signal, PPGi waveform showed better signal-to-noise ratio (SNR) and correlation (r > 0.8) when the absorption coefficient was below 10^2^ cm^−1^ where the light wavelength was close to 520 or above 590 nm, respectively. According to Eq. 3, the calculation of SaO_2_ can be derived from $R$ in theory. We investigated the theoretical relationship between $R$ and SaO_2_ for several pairs of wavelengths, where one the wavelengths in the pair was always 660 nm and the other one was, respectively, 520, 740, 750, 760, 770, 780, 790, 800 and 940 nm. As shown in Figure 5, it was found that the theoretical curve of relationship between $R$ and SaO_2_ for the combination of 520 and 660 nm is imprecise due to the very steep slope. For the other pairs the differences in the slope are insignificant.

**Figure 6:**
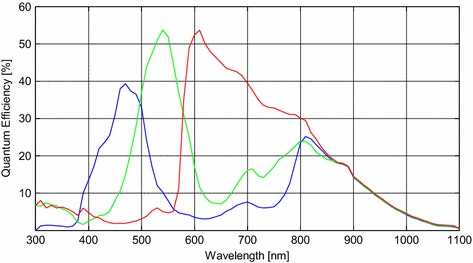
Color pixel quantum efficiency of OV9715 RGB CMOS sensor.

In addition to the two principles mentioned above, the camera spectral sensitivity should also be carefully considered. Figure 6 shows the quantum efficiency for color pixels of RGB CMOS sensor of the OV9715. Many of the commonly used cameras have similar quantum efficiency and their RGB channels are very sensitive to visible light (i.e., from 400 to 700 nm). The sensitivity is also high to light with wavelengths around 800 nm. Therefore, considering the camera sensitivity characteristics, we chose light with wavelength of 800 nm as the second illumination light.

### Sensor area and optical shunting

**Figure 7:**
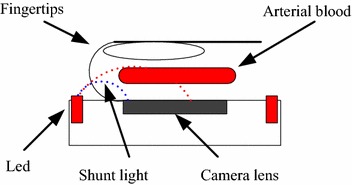
Typical setup for direct-contact PPGi measurement. The shunting induces inaccuracy in SaO_2_ estimation.

From each camera, PPGi signal was acquired by averaging the pixel values of the ROI image in every frame. In the experiment, the PPGi signal was usually acquired in an open environment. The subject’s index fingertip was put in a natural manner on the camera lens with consciously exerted force. Minor motion artifacts were inevitable [21]. With increasing the area from the sensor surface which is used to extract a PPGi signal, the SNR improves, however, another problem takes place, namely, optical shunting. As shown in Figure 7, the light beam represented by a red dot line passes through the pulsating blood vessel and determines the AC component, while the light beam represented by a blue dot line traverses through the surrounding tissue which, for the duration of the measurement, does not undergo changes and determines the DC component. Thus shunting induces unnecessary DC component and results in imprecise estimation of SaO_2_.

When the sensor is in contact with the skin, the optimal sensor area to be used for SaO_2_ calculation corresponds to the largest AC-to-DC ratio of the PPGi signal [11]. Since the DC component could be considered constant during the measurement, the AC-to-DC ratio is getting larger with increase in the AC component. The AC component will be higher with ensuring an optimal length of the light path as well as with increasing the blood flow through the fingertip vasculature. In order to avoid the negative effects of optical shunting, it was necessary to find the optimal area of the sensor surface that is to be used for calculation. For this purpose, an adaptive algorithm was developed, which, for each individual measurement, determined the optimal active sensor area size by comparing the AC-to-DC ratios of the areas formed by 100^2^, 200^2^, 300^2^, 400^2^, 500^2^, 600^2^ and 700^2^ pixels under the same wavelength light.

### Two-camera design

In our work two cameras were used. From each camera a video stream was captured, and each video stream was used as a source to extract one PPGi waveform. In terms of real-time performance, our design satisfies very well the requirements towards sampling rate. It is considered that the PPGi signal falls within the range from 0.75 to 4 Hz, while the camera frame rate is 30 Hz, which is much higher than the minimum of 8 Hz required by the Nyquist theorem [22]. Since in our approach each camera is used to capture one PPGi signal only, in terms of sampling frequency, our design outperforms the one-camera based approach shown in [11], where the effective frequency at which a PPGi signal is captured was limited to 10 Hz due to the need to allocate the resources of a single camera sensor to capture two signals. In the future, the two-camera design presented here could serve as a base for a mobile phone application for SaO_2_ measurement.

### Image pre-processing

Images were captured by each camera at a rate of 30 frames per second. After this, images were processed in the FPGA system by the video pipeline. For each image the ROI signal was separated into R, G and B components for the subsequent RGB channel processing. For each channel of the ROI the pixel values were averaged and the corresponding results were denoted as S_r_(t), S_g_(t), and S_b_(t), respectively, where t stands for the frame period. In our previous work [20], the PPGi signal of the red channel exhibited better SNR than that of the green and blue channels when the wavelength of the illumination light was above 600 nm. Thus, in the present work, two PPGi signals of the red channels were recorded. They were processed offline using MATLAB®.

### Signal analysis

According to Eqs. 2 and 3, the calculation of SaO_2_ can be derived from the AC-to-DC ratios of two PPGi signals. In conventional pulse oximetry it is assumed that the PPG signal fully reflects physiological information and does not contain artifact interferences. Thus, the AC-to-DC ratio is simply calculated using the peak-to-peak amplitude of the AC part of the PPG waveform and the DC component [21, 23]. However, the arrangement of PPGi signal measurement is different from that of PPG signal measurement which uses a plastic clamp to shield the extraneous light sources and maintain good contact with the tissue. In addition, PPGi signal is susceptible to interferences of motion artifacts, since the physical displacements influence photon propagation and, thereby, the effective optical path length. Thus, PPGi signal contains noise due to motion artifacts.

**Figure 8:**
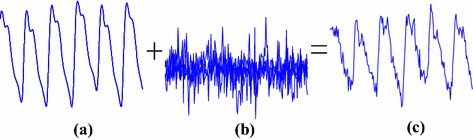
A PPGi signal (*c*) which amounts to superposition of the motion artifact (*b*) and physiological signal (*a*).

**Figure 9:**
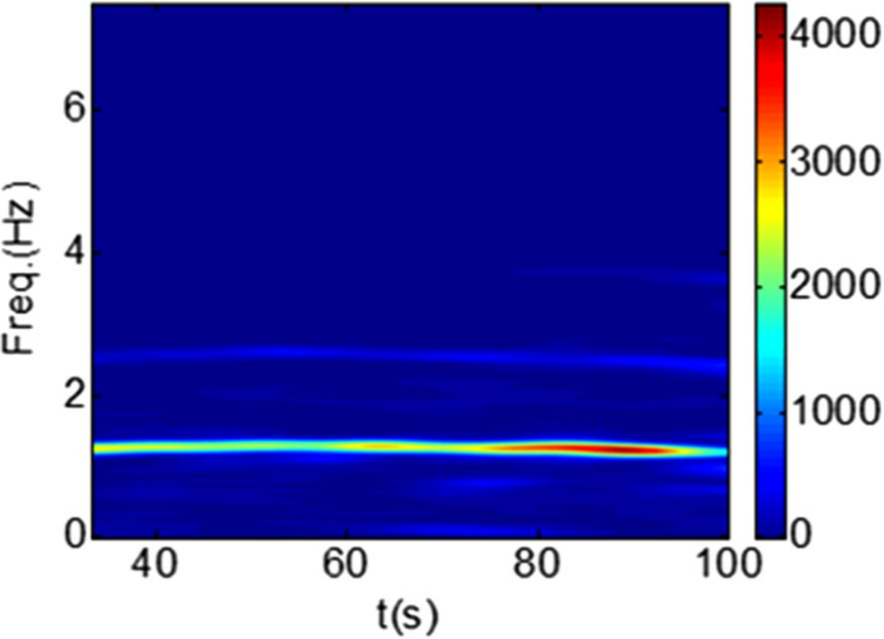
SPWVD results for a PPGi signal; the *color bar* indicates the absolute power intensity.

As shown in Figure 8, PPGi signal amounts to the superposition of motion artifacts and physiological signal. The PPGi signal shows high correlation with PPG. However, if severe morphological distortions appear in the maxima or minima regions of the PPGi signal, they may result in inaccurate estimation of SaO_2_. The frequency domain transformation of PPGi signal can reveal some fundamental information, such as HR and RR, and the power that falls within each spectral component could provide information about the amplitude of that component in the signal. Therefore, the power of AC and DC components was acquired by frequency domain transformation. To obtain values of AC and DC components over time, joint time–frequency analysis (e.g., TFR) was performed. In the TFR approach, a one-dimensional signal is converted into a two-dimensional function of time and frequency so that frequency components can be localized with a good temporal resolution. Recent PPGi studies have revealed that the SPWVD approach can allow for more reliable physiological assessment [5, 24, 25]. Compared to other TFR methods, such as the short-time Fourier transform, SPWVD allows to better characterize properties of the PPGi signals in the joint time–frequency domain [26]. Thus, the SPWVD approach was chosen to estimate the PPGi signals in our study. SPWVD is defined as:

5 $$ SPWVD(t,f) = \int\limits_{l = - P + 1}^{P - 1} {h(\tau )} \int\limits_{m = - Q + 1}^{Q - 1} {g(s - t)x\left( {s + \frac{\tau }{2}} \right)x^{*} \, \times \,\left( {s - \frac{\tau }{2}} \right)} e^{ - 2j\pi f\tau } dsd\tau $$

where $x(s)$ and $x^{*} (s)$, are the instantaneous auto-correlation functions, $g(s)$ is a Gaussian smoothing window of 2Q − 1 length used in time direction while $h(\tau )$ is a Hamming smoothing window of length 2P − 1 used for frequency smoothing. Figure 9 shows the SPWVD results for a PPGi signal where the color bar indicates the absolute power intensity.

The main drawback of the SPWVD is the presence of cross terms, which can be attenuated by time and frequency filtering. In this study, we preferred to use the SPWVD to track the rapid changes in the frequency and amplitude of the PPGi signal. The time smoothing was performed using a 5.05 s Gaussian window, while a 10.05 s Hamming window was used for frequency smoothing. The exploration of PPGi signals in specific time-varying frequency bands makes the problem of cross-terms attenuation less serious.

### Estimation of SaO_2_

**Figure 10:**
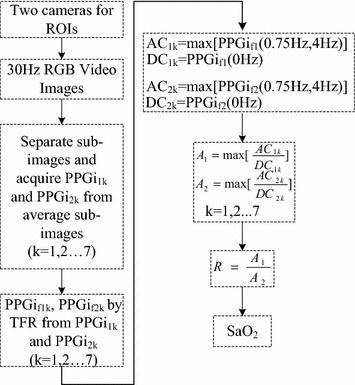
A block diagram which illustrates the calculation of SaO_2_.

The frequency of PPGi signal typically falls within the range of 0.75–4 Hz (which corresponds to 45–240 beats min^−1^). In our work, the frequency component which comprises the largest part of signal’s power was considered as the AC component and its time-domain amplitude was derived from its power in the time–frequency representation. The DC component of the PPGi signal was determined by the power allocated at 0 Hz in the time–frequency representation. Thus, $R$ was acquired according to Eq. 2. Figure 10 shows a block diagram which illustrates the calculation of SaO_2_.

### Statistical analysis

The statistical analysis was performed using the SPSS software package (version 17.0 from IBM). The mean deviation and standard deviation (SD) of the differences were calculated. The correlation between $R$ and SaO_2_ was analyzed using the correlation coefficient $r$, wherein absolute value of $r$ > 0.8 was considered as close correlation between $R$ and SaO_2_. The linear regression line was determined using the least-squares method.

## Results

The skin temperature at the measurement sites was measured at the beginning and at the end of the experimental procedure for each subject and was presented in the form of mean ± SD. At the beginning and at the end of the experiment, finger temperature was 29.8 ± 2.0 and 30.4 ± 2.1°C, respectively. During the experiment there was no significant change in the skin temperature at the measurement sites (p = 0.81).

For each of the 12 participants, PPGi signals from two cameras were simultaneously recorded and each camera operated under illumination light of different wavelength. The correlation between $R$ calculated by the proposed method and SaO_2_ acquired by the conventional pulse oximetry was calculated. The linear regression curve between $R$ and SaO_2_ was acquired.

**Figure 11:**
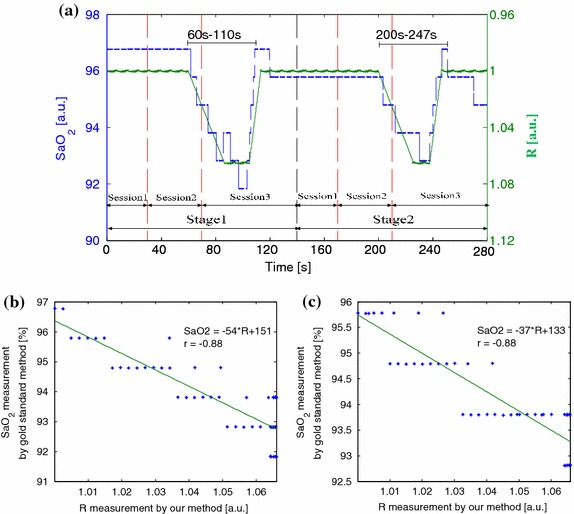
**a** Comparison of R measured by the proposed method and SaO_2_ measured by the gold standard method in two stages from a male subject aged 30 years (S01 in Table 1). **b** A correlation analysis and linear regression line for SaO_2_ and R from 60 to 110 s of the first stage. **c** A correlation analysis and linear regression line for SaO_2_ and R from 200 to 247 s of the second stage.

Figure 11a shows a comparison of R measured by the proposed method and SaO_2_ measured by the gold standard method from a male subject aged 30 years (S01 in Table 1). For all the subjects, the experiment was arranged in two stages which included three identical sessions. In the second session of every stage, the subject was asked to hold his/her breath. It was found that SaO_2_ did not decrease immediately. After around 30 s SaO_2_ reached the level of 92%. In the third session, the subject restored normal breathing, and SaO_2_ restored the original level after 30 s. In the first and second stages, the SaO_2_ estimation from our method showed good correlation with that obtained by the gold standard, especially in the time from 60 to 110 s and 200 to 247 s. Figure 11b shows the correlation analysis and linear regression line for SaO_2_ and R from 60 to 110 s of the first stage. Figure 11c shows the correlation analysis and linear regression line for SaO_2_ and R from 200 to 247 s of the second stage. The both correlation coefficients have absolute values of approx. 0.88. The slope of the linear regression was always negative because SaO_2_ is inversely-proportional to R.

**Table 1:** The linear regressions between SaO_2_ and R obtained for the experiment in which 12 subjects were tested in 2 stages

Subject	$SaO_{2} = \alpha \cdot R + \beta$			
	Stage 1		Stage 2	
	$\alpha$	$\beta$	$\alpha$	$\beta$
S01	−54	151	−37	133
S02	−45	141	−42	137
S03	−50	148	−40	138
S04	−56	153	−39	137
S05	−48	146	−30	128
S06	−43	140	−37	133
S07	−40	137	−35	134
S08	−43	141	−30	125
S09	−46	141	−42	137
S10	−48	146	−37	133
S11	−60	156	−44	141
S12	−44	141	−33	130

Table 1 shows the linear regression between SaO_2_ and R in another experiment which included two stages. In the second stage, larger contact force was applied than that in the first stage. Even though the y-intercept $\alpha$ and slope $\beta$ in the first and second stages were different, the differences were insignificant (p = 0.360 and p = 0.288 for $\alpha$ and $\beta$, respectively).

## Discussion and limitations

SaO_2_ measurement is based on the principle of pulse oximetry that relies on the different absorption spectra of HbO_2_ and Hb and on the assumption that the PPG waveform reflects changes in arterial blood volume. A novel technology, namely PPGi technology, allows using a camera in the role of a sensor to detect PPG signal and could be used for estimation of SaO_2_. In our experiment the accuracy of SaO_2_ estimation was influenced by many factors. In contrast to the conventional method, we set two different body sites (i.e., the index and thumb fingertips) as ROIs. Two different contact forces between body sites and camera lens were applied during the two stages of the experiment. To our knowledge, this is the first study to measure SaO_2_ based on two different body sites and using cameras as sensors. The results showed good correlation (|r| > 0.8) with the reference.

### Theoretical standpoint

In the 1970s, the technique of pulse oximetry was invented. It allowed obtaining an estimate of SaO_2_ in a non-invasive manner by measuring PPG signals at two different wavelengths [1]. HbO_2_ and Hb have significantly different absorption coefficients for light in the wavelength range from 500 to 1,000 nm. The feasibility of measuring SaO_2_ remotely, using light reflected by the skin and a camera in the role of a sensor was first discussed in the literature around 2005 by Wieringa et al. [7]. They analyzed SaO_2_ distribution within the skin tissue of the arm, based upon detection of a two-dimensional matrix of spatially resolved optical plethysmographic signals at different wavelengths (i.e., 660, 810 and 940 nm). The heartbeat-related and respiration-correlated PPGs captured at three wavelengths using a remote camera showed potential for the estimation of the SaO_2_. In 2007, Humphreys et al. [8] used a CMOS camera for capturing the light reflected from the inner arm of ten volunteers, adjacent to an area illuminated by an array of LEDs with wavelengths of 760 and 880 nm. This allowed two multiplexed PPG waveforms to be captured simultaneously at a rate of 16 fps. The agreement between estimates of heart rate derived from the camera images and from a conventional pulse oximeter was shown to be excellent. Some teams investigated the feasibility of estimating oxygen saturation based on the principle of pulse oximetry and using camera images captured under light illumination of two different wavelengths. For example, Kong et al. [9] demonstrated an accurate video-based method for non-contact oxygen saturation measurement using ambient light with its respective visible wavelength spectrum in the range of 660 and 520 nm. Scully et al. [10] and Tarassenko et al. [27] both presented a camera-based method for estimation of SaO_2_. Measurements of relative SaO_2_ were performed by comparing PPGi signals of the red and blue bands. The combination where one of the wavelengths was from the red band and the other was from the blue band was found to conform the two requirements for absorption by Hb and HbO_2_ and it was also found to outperform the pair 660–940 nm. In this case, the SaO_2_ can be acquired as follows:

6 $$ SaO_{2} = A - B \times \frac{{{{AC_{RED} } \mathord{\left/ {\vphantom {{AC_{RED} } {DC_{RED} }}} \right. \kern-0pt} {DC_{RED} }}}}{{{{AC_{BLUE} } \mathord{\left/ {\vphantom {{AC_{BLUE} } {DC_{BLUE} }}} \right. \kern-0pt} {DC_{BLUE} }}}} $$

The parameters A and B for each subject were determined according to Eq. 6 where the SaO_2_ value was substituted with values obtained by a commercial pulse oximeter. The best-fit linear equation was determined using the Matlab Curve Fitting Toolbox.

According to Beer–Lambert law, we can derive:

7 $$ \frac{{I_{AC} }}{{I_{DC} }} = (\varepsilon_{{HbO_{2} }} c_{{HbO_{2} }} + \varepsilon_{Hb} c_{Hb} ) \cdot \Delta L $$

where I_AC_ is the pulsatile component (determined by the pulsations of arterial blood), and I_DC_ is the constant component (determined by venous blood, tissue, bones, etc.) of the measured PPG signal for the specified wavelengths $\lambda$, c denotes the corresponding concentration at the specific wavelength, and $\Delta L$ is the increased length of the path which the light passes through the arterial blood.

8 $$ \left\{ \begin{aligned}{l} D_{{\lambda_{1} }} = \frac{{I_{AC}^{{\lambda_{1} }} }}{{I_{DC}^{{\lambda_{1} }} }},\quad D_{{\lambda_{2} }} = \frac{{I_{AC}^{{\lambda_{2} }} }}{{I_{DC}^{{\lambda_{2} }} }} \hfill \\ \frac{{D_{{\lambda_{1} }} }}{{D_{{\lambda_{2} }} }} = \frac{{{{I_{AC}^{{\lambda_{1} }} } \mathord{\left/ {\vphantom {{I_{AC}^{{\lambda_{1} }} } {I_{DC}^{{\lambda_{1} }} }}} \right. \kern-0pt} {I_{DC}^{{\lambda_{1} }} }}}}{{{{I_{AC}^{{\lambda_{2} }} } \mathord{\left/ {\vphantom {{I_{AC}^{{\lambda_{2} }} } {I_{DC}^{{\lambda_{2} }} }}} \right. \kern-0pt} {I_{DC}^{{\lambda_{2} }} }}}} = \frac{{(\varepsilon_{{HbO_{2} }}^{{\lambda_{1} }} C_{{HbO_{2} }} + \varepsilon_{Hb}^{{\lambda_{2} }} C_{Hb} )}}{{(\varepsilon_{{HbO_{2} }}^{{\lambda_{2} }} C_{{HbO_{2} }} + \varepsilon_{Hb}^{{\lambda_{2} }} C_{Hb} )}} \times \Delta_{1} \hfill \\ \Delta_{1} = \frac{{\Delta L_{{\lambda_{1} }} }}{{\Delta L_{{\lambda_{2} }} }} \hfill \\ \end{aligned} \right. $$

Using Eq. 8, the theoretical calibration curve for the calculation of functional SaO_2_ can be defined as follows:

9 $$ SaO_{2} = \frac{{\varepsilon_{Hb}^{{\lambda_{2} }} \cdot \left( {{{\frac{{D_{{\lambda_{1} }} }}{{D_{{\lambda_{2} }} }}} \mathord{\left/ {\vphantom {{\frac{{D_{{\lambda_{1} }} }}{{D_{{\lambda_{2} }} }}} {\Delta_{1} }}} \right. \kern-0pt} {\Delta_{1} }}} \right) - \varepsilon_{Hb}^{{\lambda_{1} }} }}{{(\varepsilon_{{HbO_{2} }}^{{\lambda_{1} }} - \varepsilon_{Hb}^{{\lambda_{1} }} ) - (\varepsilon_{{HbO_{2} }}^{{\lambda_{2} }} - \varepsilon_{Hb}^{{\lambda_{2} }} ) \cdot \left( {{{\frac{{D_{{\lambda_{1} }} }}{{D_{{\lambda_{2} }} }}} \mathord{\left/ {\vphantom {{\frac{{D_{{\lambda_{1} }} }}{{D_{{\lambda_{2} }} }}} {\Delta_{1} }}} \right. \kern-0pt} {\Delta_{1} }}} \right)}} \times 100\% $$

The Eq. 9 can still be abbreviated as follows:

10 $$ SaO_{2} = \alpha_{2} \cdot R + \beta_{2} $$

where the y-intercept $\beta_{2}$ and the slope $\alpha_{2}$ are empirical coefficients determined by calibration. It was proved that estimate of SaO_2_ from two different ROIs was still feasible.

### Design challenges

Karlen et al. [11] investigated the design challenges for camera oximetry, and focused especially on the considerations regarding the selection of optimal sensor surface area size for SaO_2_ calculation and avoiding the effects of optical shunting. The main challenge is the non-homogeneous distribution of light over the sensor which means that the AC-to-DC ratio is different at the different sensor surface regions. For example, as Figure 7 shows, the regions which are closer to the light source are associated with higher value of the DC component while the areas which are far from the light source are characterized with higher values of the AC component. We addressed this problem in two ways. First, the shape of the LED illuminators allows for enhancing the homogeneous distribution of the light over the sensor. Second, we compared the AC-to-DC ratios of the PPGi signals extracted when using different portions of the sensor area for the calculation. For this goal we defined several sizes which included different amount of pixels, i.e., 100^2^, 200^2^, 300^2^, 400^2^, 500^2^, 600^2^ and 700^2^ pixels. As optimal value was considered the one which ensured the largest AC-to-DC ratio of the PPGi signal.

The SaO_2_ was calculated using the AC and DC components of the PPGi signal [28]. However, the peak-to-peak value and DC bias extracted from the PPGi signal vary from cycle to cycle even when the blood saturation remains unchanged. In attempt to address this problem, Scully et al. [10] and Kong et al. [9] both applied a 10 s moving average window to the AC and DC components, however, the error from artifact noise was still present. SPWVD allows for better characterization of the PPGi signal properties in the joint time–frequency domain.

**Figure 12:**
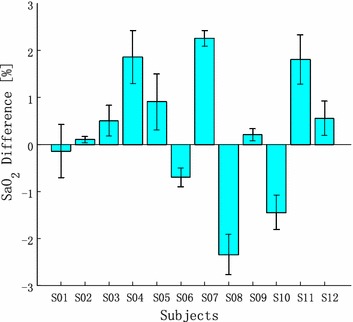
The individual mean SD of the differences between the measured and predicted values in the second stage.

Except by motion artifacts, the AC and DC components of the PPGi signal are also influenced by the inconsistency of the contact force [12]. In our previous study, the inconsistency of the contact force between the skin and camera lens also affected the AC and DC components and, respectively, the AC-to-DC ratio [20]. Figure 12 shows the individual mean ± SD of the differences between the measured and predicted values in the second stage. The estimates of SaO_2_ in the first and second stages were both highly correlated with the gold standard SaO_2_ and the coefficients in the regressions from the test were similar. However, the difference in estimation as a result of different contact forces was rather large when SaO_2_ in the second stage was predicted using the regression coefficients from the first stage.

### Limitations of the present study

Though the proposed method solved some technological challenges for calculation of SaO_2_ and its results showed a high correlation (r^2^ > 0.85) and accuracy (d = 0.5%, SD < ±2%) when compared with those of the conventional pulse oximetry in the range of 90–100%, there is an important limitation. It is mainly caused by the inconsistency of the contact force between the skin and camera lens. Different contact forces determine different calibration curves. Even though the difference of the y-intercept $\alpha$ and slope $\beta$ for different contact forces was insignificant (p = 0.360 and p = 0.288 for $\alpha$ and $\beta$, respectively), it induces error. The plastic clamps used in the conventional pulse oximetry are designed in a way to ensure constant contact force. We acquired the empirical calibration curve well. As shown in Figures 11 and 12, the additionally applied force changes the coefficient of the regression line and in this case it is difficult to determine the empirical curve. The SaO_2_ calibration remains an open challenge. However, when the contact force is constant, our method ensures high correlation between SaO_2_ and R.

## Conclusions

Pulse oximetry plays an important role in monitoring the health of patients and is widely used in intensive care, operation rooms, emergency care, patient transport, general wards, birth and delivery, neonatal care, sleep laboratories, and home care. Smartphones with two micro cameras are very popular among the general public. This fact motivated us to develop a method for SaO_2_ estimation based on two cameras and direct skin-to-camera contact. We took into account all design challenges. The results show high correlation and accuracy when compared with those of the conventional pulse oximetry. Our method is suitable for implementation in smartphones equipped with two cameras and could allow multi-parameter physiological measurement in a pervasive environment.
